# Sirtuin3 Dysfunction Is the Key Determinant of Skeletal Muscle Insulin Resistance by Angiotensin II

**DOI:** 10.1371/journal.pone.0127172

**Published:** 2015-05-19

**Authors:** Daniela Macconi, Luca Perico, Lorena Longaretti, Marina Morigi, Paola Cassis, Simona Buelli, Norberto Perico, Giuseppe Remuzzi, Ariela Benigni

**Affiliations:** 1 IRCCS—Istituto di Ricerche Farmacologiche ^“^Mario Negri”, Centro Anna Maria Astori, Science and Technology Park Kilometro Rosso, Via Stezzano 87, 24126 Bergamo, Italy; 2 IRCCS—Istituto di Ricerche Farmacologiche ^“^Mario Negri, Clinical Research Center for Rare Diseases “Aldo e Cele Daccò”, Via Camozzi 3, 24020 Ranica, Bergamo, Italy; 3 Unit of Nephrology and Dialysis, Azienda Ospedaliera Papa Giovanni XXIII, Piazza OMS 1, 24127 Bergamo, Italy; University Medical Center Utrecht, NETHERLANDS

## Abstract

**Background:**

Angiotensin II promotes insulin resistance. The mechanism underlying this abnormality, however, is still poorly defined. In a different setting, skeletal muscle metabolism and insulin signaling are regulated by Sirtuin3.

**Objective:**

Here, we investigate whether angiotensin II-induced insulin resistance in skeletal muscle is associated with Sirtuin3 dysregulation and whether pharmacological manipulation of Sirtuin3 confers protection.

**Study Design:**

Parental and GLUT4-myc L6 rat skeletal muscle cells exposed to angiotensin II are used as *in vitro* models of insulin resistance. GLUT4 translocation, glucose uptake, intracellular molecular signals such as mitochondrial reactive oxygen species, Sirtuin3 protein expression and activity, along with its downstream targets and upstream regulators, are analyzed both in the absence and presence of acetyl-L-carnitine. The role of Sirtuin3 in GLUT4 translocation and intracellular molecular signaling is also studied in Sirtuin3-silenced as well as over-expressing cells.

**Results:**

Angiotensin II promotes insulin resistance in skeletal muscle cells via mitochondrial oxidative stress, resulting in a two-fold increase in superoxide generation. In this context, reactive oxygen species open the mitochondrial permeability transition pore and significantly lower Sirtuin3 levels and activity impairing the cell antioxidant defense. Angiotensin II-induced Sirtuin3 dysfunction leads to the impairment of AMP-activated protein kinase/nicotinamide phosphoribosyltransferase signaling. Acetyl-L-carnitine, by lowering angiotensin II-induced mitochondrial superoxide formation, prevents Sirtuin3 dysfunction. This phenomenon implies the restoration of manganese superoxide dismutase antioxidant activity and AMP-activated protein kinase activation. Acetyl-L-carnitine protection is abrogated by specific Sirtuin3 siRNA.

**Conclusions:**

Our data demonstrate that angiotensin II-induced insulin resistance fosters mitochondrial superoxide generation, in turn leading to Sirtuin3 dysfunction. The present results also highlight Sirtuin3 as a therapeutic target for the insulin-sensitizing effects of acetyl-L-carnitine.

## Introduction

Insulin resistance is a key component of metabolic syndrome, a cluster of hypertension, diabetes mellitus, dyslipidemia, and chronic inflammation, observed frequently in subjects who are obese or have a family history of diabetes, and renal or cardiovascular disease [1, 2]. Metabolic syndrome affects approximately 30% of adults in Europe [3], but this percentage is rapidly increasing worldwide, particularly in emerging countries, indicating there may be a diabetes epidemic in the next few decades [4]. Identifying the mechanistic origin of insulin resistance remains a major objective in designing innovative treatments.

One of several insulin-sensitive tissues, skeletal muscle plays a well-established role in maintaining glucose homeostasis and in regulating whole body carbohydrate metabolism. Skeletal muscle is the major site of insulin-mediated glucose uptake [5, 6] and insulin resistance in this tissue causes metabolic syndrome [7] and is recognized as the initial metabolic defect for the development of type 2 diabetes [8].

Multiple factors contribute to insulin resistance, involving interacting mechanisms such as ectopic lipid accumulation, endoplasmic reticulum stress, pro-inflammatory response, and, importantly, the activation of the renin-angiotensin system (RAS) [9, 10]. The major effector of RAS is angiotensin II (Ang II)—a critical promoter of insulin resistance because of its effect on insulin receptors and downstream signaling, which results in desensitization to insulin in metabolic tissues [11]. The intracellular mechanism through which Ang II impairs insulin signaling in skeletal muscle cells and in isolated skeletal muscles is thought to be NADPH-oxidase-derived reactive oxygen species (ROS) [12–14]. Based on the evidence that insulin resistance is associated with mitochondrial dysfunction [15–17], researchers have increasingly focused on the possibility that insulin resistance and metabolic syndrome are dependent on altered mitochondrial oxidation and reactive oxygen species production [16, 18]. Chronic Ang II infusion causes glucose intolerance and mitochondrial abnormalities in skeletal muscles of healthy mice together with increased mitochondrial ROS generation [19]. In a model of insulin resistance associated with excessive RAS activation, mitochondrial abnormalities are observed in the liver, skeletal muscles and the myocardium [15]. Whether or not angiotensin II-induced insulin resistance is causally linked to mitochondrial ROS generation and the downstream target(s) of this phenomenon remains to be ascertained.

We previously described a link between Ang II and Sirtuin3 (Sirt3), the primary mitochondrial NAD^+^-dependent deacetylase that regulates energy and redox homeostasis, protecting mitochondria from oxidative damage [20]. Mice deficient in the *Agtr1* gene—which encodes the Ang II type 1a receptor—showed multiple organ protection from age-induced oxidative stress and preservation of mitochondrial numbers in renal tubules linked with Sirt3 upregulation [21].

Modulators of mitochondrial metabolism have been studied in an attempt to ameliorate metabolic abnormalities associated with mitochondrial dysfunction in diverse experimental and human conditions. One of these is the recent finding that acetyl-L-carnitine (ALCAR) [22] ameliorated glucose tolerance and insulin resistance in subjects with a cluster of risk factors for diabetes mellitus and cardiovascular disease [23].

In the present study, we investigate whether angiotensin II-induced insulin resistance in skeletal muscle is associated with Sirt3 dysregulation and whether pharmacological manipulation of Sirt3 confers protection.

## Materials and Methods

### Cell culture and incubation

The rat skeletal muscle myoblast cell line L6 (ATCC, LGC Standards S.r.l., Sesto San Giovanni (Mi), Italy) was cultured in DMEM (Sigma-Aldrich, Saint Louis, MO) supplemented with L-glutamine (2 mM), 10% FCS, penicillin (100 U/mL) and streptomycin (100 μg/mL) in a humidified atmosphere of 5% CO_2_ at 37°C [13]. L6 GLUT4-myc myoblast cell line [24], kindly provided by Dr. Amira Klip (Cell Biology Program, The Hospital for Sick Children, Toronto, Ontario, Canada), was cultured in α-MEM (GIBCO-Invitrogen, Gaithesburg, MA) supplemented with 10% FCS, blasticidin-HCl (2 mg/mL), penicillin (100 U/mL) and streptomycin (100 μg/mL) in a humidified atmosphere of 5% CO_2_ at 37°C. To induce differentiation, parental and L6 GLUT4-myc myoblasts were maintained in DMEM supplemented with 2% horse serum and antibiotics for 12 days or in α -MEM supplemented with 2% FCS and antibiotics for 8 days, respectively, and used at the myotube stage (60%) until the 15^th^ passage.

Myotubes were deprived of serum for 3 h at 37°C before experimentation. For GLUT4 translocation assessment, cells were incubated with low glucose (1.0 g/L) DMEM or α-MEM in the absence (control) and presence of 100 nM Ang II (Sigma-Aldrich) 24 h before and during 30-min stimulation with 100 nM insulin (Sigma-Aldrich). The dose of 100 nM insulin, a submaximal dose of the hormone, is typically used to study insulin sensitivity in acutely stimulated L6 myotubes [25]. For glucose uptake, the medium was replaced by glucose-free Hepes-buffered saline during incubation with insulin supplemented for the final 10 min with D-2-deoxy-[^3^H]-glucose (10 μM, 2 μCi/mL, Perkin Elmer, Italia, Monza Italy).

ALCAR (0.6 mM, Sigma Tau, Rome, Italy), manganese(III)tetrakis (4-benzoic acid)porphyrin (MnTBAP) (0.1 mM, Santa Cruz Biotechnology, Santa Cruz, CA), 5-aminoimidazole-4-carboxamide-1β-D-ribofuranoside (AICAR) (500 μM, Toronto Research Chemicals Inc, Ontario, Canada), cyclosporin A (CsA, 1 μM, Novartis Farma S.p.A., Origgio, Italy) were added to parental or L6 GLUT4-myc myotubes 1 h before Ang II and maintained throughout the experiment. Compound C 6-[4-(2-Piperidin-1-ylethoxy)phenyl]-3-pyridin-4-ylpyrazolo[1,5-a] pyrimidine (10 μM, Sigma-Aldrich), a specific inhibitor of AMP-activated protein kinase (AMPK), was added to unstimulated parental or L6 GLUT4-myc myotubes for the same incubation times used for Ang II-treated cells.

### 2-deoxyglucose uptake

The assay was performed in L6 myotubes as described by Yonemitsu et al. [26]. Specific 2-deoxyglucose uptake was expressed as pmol/min/mg protein from determination performed at least in triplicate.

### Subcellular fractionation

The subcellular fractionation of L6 myotubes was performed as described by Mitsumoto and Klip [27] with slight modifications. After incubations, cells were gently scraped and incubated in hypotonic lysis buffer (10 mM Tris-HCl pH 7.4, 2 mM EDTA, 200 μM PMSF, 1 mM benzamidine, 10 μg/mL pepstatin and 10 μg/mL leupeptin) for 20 min on ice and then lysed by sonication. An aliquot of the total cell lysate was saved for western blot analysis of the total GLUT4 and the remaining sample was centrifuged at 1,000 g for 10 min at 4°C to remove nuclei and unbroken cells. The supernatant was centrifuged at 31,000 g for 60 min to pellet crude plasma membrane (CPM).

### Measurement of GLUT4-myc translocation

Detection of GLUT4-myc on the cell surface of intact L6 GLUT4-myc myotubes was assessed using a colorimetric-based assay [24].

### Mitochondrial ROS production

Mitochondrial ROS were measured using MitoSOX Red, a live-cell permeant mitochondrial superoxide (O_2_^•-^) indicator (Molecular Probes, Invitrogen, Life Technologies, Milan, Italy) (5 μM) added to control or Ang II-treated cells for the last h-incubation. Cells were collected by tripsinization, washed, and mitochondrial superoxide was determined by FACS (FACS Canto, BD Biosciences, Milan, Italy). MitoSOX Red was excited by laser at 510 nm and data collected at FSC, SSC, 580 nm (FL2) channel. Data were expressed as mean intensity of MitoSOX fluorescence and % of MitoSOX fluorescent cells.

### Mitochondrial membrane potential (ΔΨ)

ΔΨ was studied in L6 myotubes exposed to JC-1 fluorescent dye (5,5′,6,6′- tetrachloro- 1,1′,3,3′- tetra-ethyl-benzimidazolyl-carbocyanine iodide, Invitrogen) (5 μM) for the last 30 min-incubation at 37°C, 5% CO_2_. JC-1 exhibits potential-dependent accumulation in mitochondria and forms aggregates in polarized and healthy mitochondria emitting a red fluorescence (~580 nm). On the contrary, JC-1 does not aggregate and exists as a monomer with green fluorescence (~530 nm) when scattered in the cytosol, indicating ΔΨ loss. Nuclei were stained with 4',6-diamidino-2-phenylindole (DAPI) (Sigma-Aldrich). Samples were examined under an Apotome fluorescent microscope (Zeiss, Jena, Germany).

### Immunocytochemistry

Paraformaldehyde-fixed cells were incubated with Triton 0.3% and, after blocking, with a rabbit monoclonal anti-Sirt3 antibody (1:100, Cell Signaling Technology Inc., Danvers, MA), followed by a goat anti-rabbit Cy3-conjugated secondary antibody (Jackson ImmunoResearch Laboratories, Baltimore Pike, PA). Nuclei were stained with DAPI (Sigma-Aldrich). Sirt3 staining was examined under confocal laser scanning microscopy (LS 510 Meta, Zeiss). Sirt3 protein expression was quantified in 10 randomly acquired fields per sample. Specifically, the area corresponding to the Sirt3 staining was measured in pixels^2^ using Image J 1.40g software and normalized for the number of DAPI-positive nuclei.

### Protein extraction and mitochondria isolation

L6 myotubes were harvested in lysis buffer for phosphorylated protein analysis (50 mM Hepes pH 7.4, 150 mM NaCl, 1mM EDTA, 1mM EGTA, 0.5 mM NaVO_4_, 10 mM Na floride, 1 mM β-glycerophosphate, 20 mM H_3_NaO_7_P_2_, 10% glycerol, 1% TritonX-100, supplemented with protease inhibitor cocktail Sigma Aldrich), lysed by sonication and centrifuged 15,000 g for 15 min at 4°C to remove detergent-insoluble material. Mitochondria were isolated from L6 myotubes using Qproteome Mitochondria Isolation Kit (Qiagen S.r.l., Milan, Italy) according to the manufacturer’s protocol. Isolated mitochondria were solubilized in mitochondrial storage buffer (150 mM NaCl, 50 mM Tris-HCl pH 7.4, 10 mM nicotinamide, 500 nM tricostatin A, 1% *n*-dodecyl-β-maltoside). Mitochondrial and total protein concentration was determined by DC assay (Bio-Rad Laboratories, Hercules, CA, USA).

### Western blot analysis

Whole cells, subcellular fractions or isolated mitochondria from L6 myotubes were analyzed. Proteins were separated on SDS-PAGE under reducing conditions and transferred to PVDF membranes (Bio-Rad Laboratories) that were incubated overnight at 4°C with primary antibodies. The following antibodies were used: mouse monoclonal anti-GLUT4 antibody (1:1000; Abcam, Cambridge, UK), goat anti-Sirt3 antibody (1:200; Santa Cruz Biotechnology), rabbit anti-phospho-AMPKα (Thr172) or anti-total AMPKα antibody (1:1000; Cell Signaling), rabbit anti-acetylated-lysine antibody (1:1000; Cell Signaling), rabbit anti-VDAC antibody (1:1000; Cell Signaling), sheep anti-superoxide dismutase (Mn Enzyme) antibody (1:2000, Calbiochem-EMD Millipore Corporation, Billerica, MA), and mouse anti-tubulin antibody (1:1000; Sigma Aldrich). The signal was visualized using the corresponding HRP-conjugated secondary antibodies and ECL Western blotting Detection Reagent (Pierce, Life Technologies). Bands were quantified by densitometry using the Image J 1.40g software.

### Real time PCR

The RNA of L6 myotubes or skeletal muscle tissue (2 μg), purified by Trizol reagent, was reverse-transcribed using the SuperScript II First Strand Synthesis System (Invitrogen). Transcript levels of target and housekeeping genes were assessed with an ABI 7300 Real Time PCR System using SYBR Green Master Mix (Applied Biosystems, Warrington, UK) with the following primers: rat manganese superoxide dismutase (*MnSOD*), forward 5'-GGGCTGGCTTGGCTTCA-3', reverse 5'-AGCAGGCGGCAATCTGTAA-3'; rat nicotinamide phosphoribosyltransferase (*NAMPT*), forward 5’-GCAGAAGCCGAGTTCAACATCCT-3’, reverse 5’-ACTTTGCTTGTGTTGGGTGGGT-3’; rat *Sirt3*, forward 5’-CAAGGTTCTTACTACATGTGGCTGATT-3, reverse 5’-GGCACTGATTTCTGTACCGATTC-3’; rat *GAPDH*, forward 5’- TCATCCCTGCATCCACTGGT-3’, reverse 5’-CTGGGATGACCTTGCCCAC-3’. Data were analyzed using the 2^-ΔΔCT^ method and presented as fold changes relative to unstimulated cells (Control).

### SOD activity assay

SOD activity in isolated mitochondria was measured using a Colorimetric Activity kit (Arbor Assays, Ann Arbor, MI).

### Sirt3 silencing

L6 GLUT4-myc and parental L6 myotubes were transfected with ON-TARGET plus SMART pool Sirt3 (100 pmol) duplex for the target sequence NM_001106313, or with control nontarget siRNA (Ambion, Silencer Select Negative Control #2siRNA) using Lipofectamine 2000 reagent (Invitrogen) according to the manufacturer’s protocol. Forty-eight h after transfection, L6 GLUT4-myc myotubes were used for GLUT4 translocation assessment. Western blot analysis of pAMPK/AMPK was performed in L6 cells 96 h after transfection.

### Sirt3 overexpression

L6 myotubes were transfected with plasmid DNA (2,5 μg GFP-tagged pCMV-hSirt3, Origene Technologies) using Lipofectamine 2000 (Invitrogen, Life Technologies) as described in the manufacturer’s instructions. Afterwards cells were exposed to medium with or without Ang II for 15 h.

### Statistical analysis

Results are expressed as mean ± SE. Data analysis was performed using the computer software Prism (GraphPad Software, Inc. San Diego USA). Comparisons were made by ANOVA with the Bonferroni post hoc test or unpaired Student’s t test as appropriate. Statistical significance was defined as P < 0.05.

## Results

### Ang II promotes insulin resistance via mitochondrial ROS, which is reverted by ALCAR

**Fig 1:**
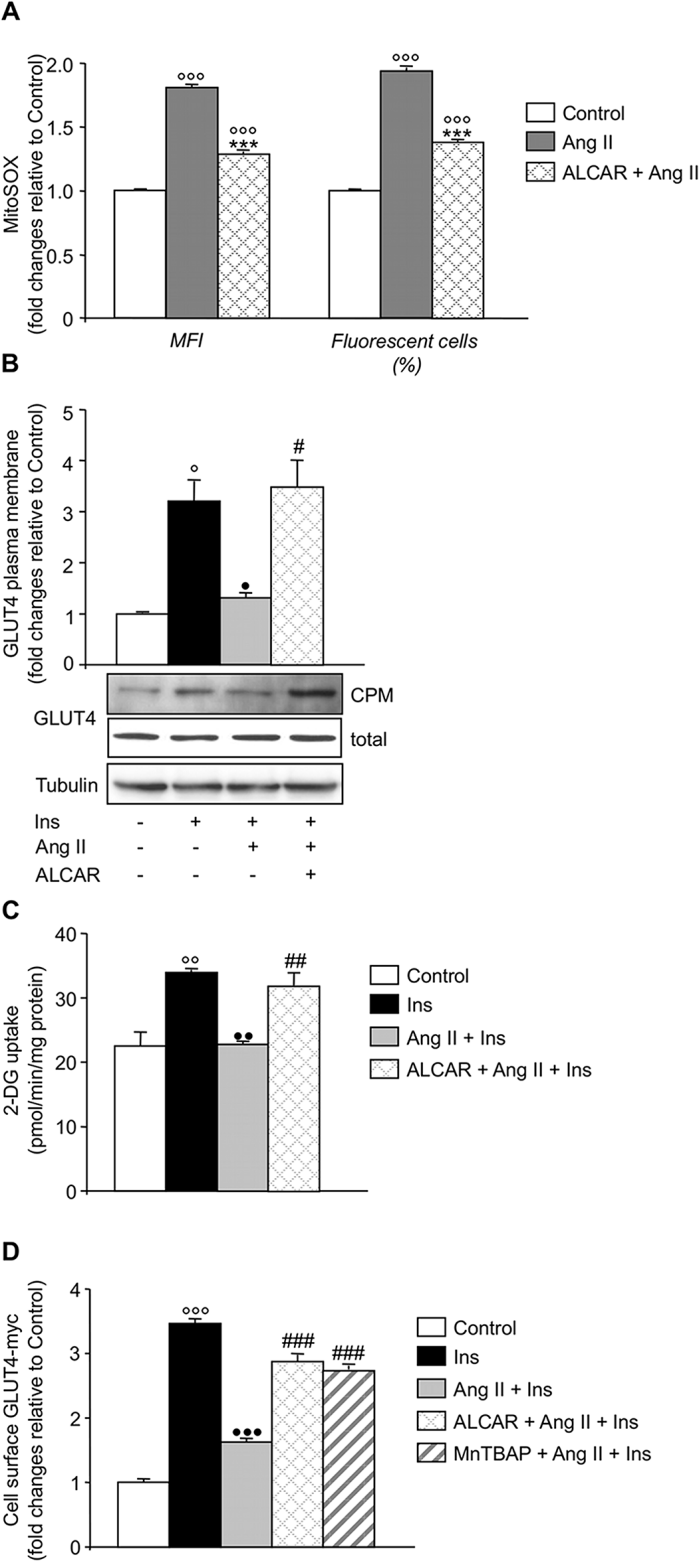
Ang II promotes insulin resistance via mitochondrial ROS. Effect of Ang II on (A) mitochondrial O_2_^•^ generation, (B) insulin (Ins)-stimulated GLUT4 transport (densitometric analysis, top, and representative western blot, bottom) and (C) Ins-stimulated 2-deoxyglucose (2-DG) uptake in L6 myotubes in the absence and presence of ALCAR (0.6 mM). MFI, mean fluorescence intensity; CPM, crude plasma membrane. (D) Effect of Ang II on surface GLUT4-myc density in Ins-stimulated cells in the absence and presence of ALCAR or MnTBAP (0.1 mM). Results are mean ± SE (n = 3, A-C; n = 6, D). °P < 0.05, °°P < 0.01, °°°P < 0.001 *vs*. Control; •P < 0.05, ••P < 0.01, •••P < 0.001 *vs*. Ins; ***P < 0.001 *vs*. Ang II; #P < 0.05, ##P < 0.01, ###P < 0.001 *vs*. Ang II + Ins.

Ang II triggered mitochondrial ROS generation in L6 myotubes as documented by a two-fold increase over controls both in the cellular production of O_2_^•^ (MitoSOX, mean fluorescence intensity-MFI-) and in the percentage of cells undergoing oxidative stress (Fig 1A). This effect was associated with a 59% inhibition of insulin-stimulated GLUT4 translocation to the cell plasma membrane (Fig 1B) and the reduction of cell glucose uptake (Fig 1C). Treatment with ALCAR significantly reduced excessive mitochondrial O_2_^•^ and prevented Ang II-induced insulin resistance (Fig 1A–1C). To further confirm results obtained in parental L6 myotubes, L6 muscle cells stably expressing GLUT4 tagged on an extracellular domain with a myc epitope were used. Ang II also impaired insulin-stimulated GLUT4 transport in GLUT4-myc L6 myotubes, which was restored by ALCAR (Fig 1D). Similarly, MnTBAP, a mitochondria-penetrating SOD mimetic, rescued GLUT4-myc cells from Ang II-induced insulin resistance confirming that scavenging mitochondrial O_2_^•^ improved insulin sensitivity (Fig 1D). ALCAR alone did not affect the insulin sensitivity of L6 myotubes evaluated as GLUT4 translocation (cell surface GLUT4-myc fold changes, ALCAR + Ins 3.08 ± 0.23 *vs*. Ins 3.47 ± 0.06). Notably, Ang II alone significantly reduced apical expression of GLUT4 in GLUT4-myc L6 myotubes, an effect that was prevented by ALCAR (S1 Fig).

### Ang II via ROS inhibits Sirt3 and impairs mitochondrial antioxidant defense, both of which are restored by ALCAR

**Fig 2:**
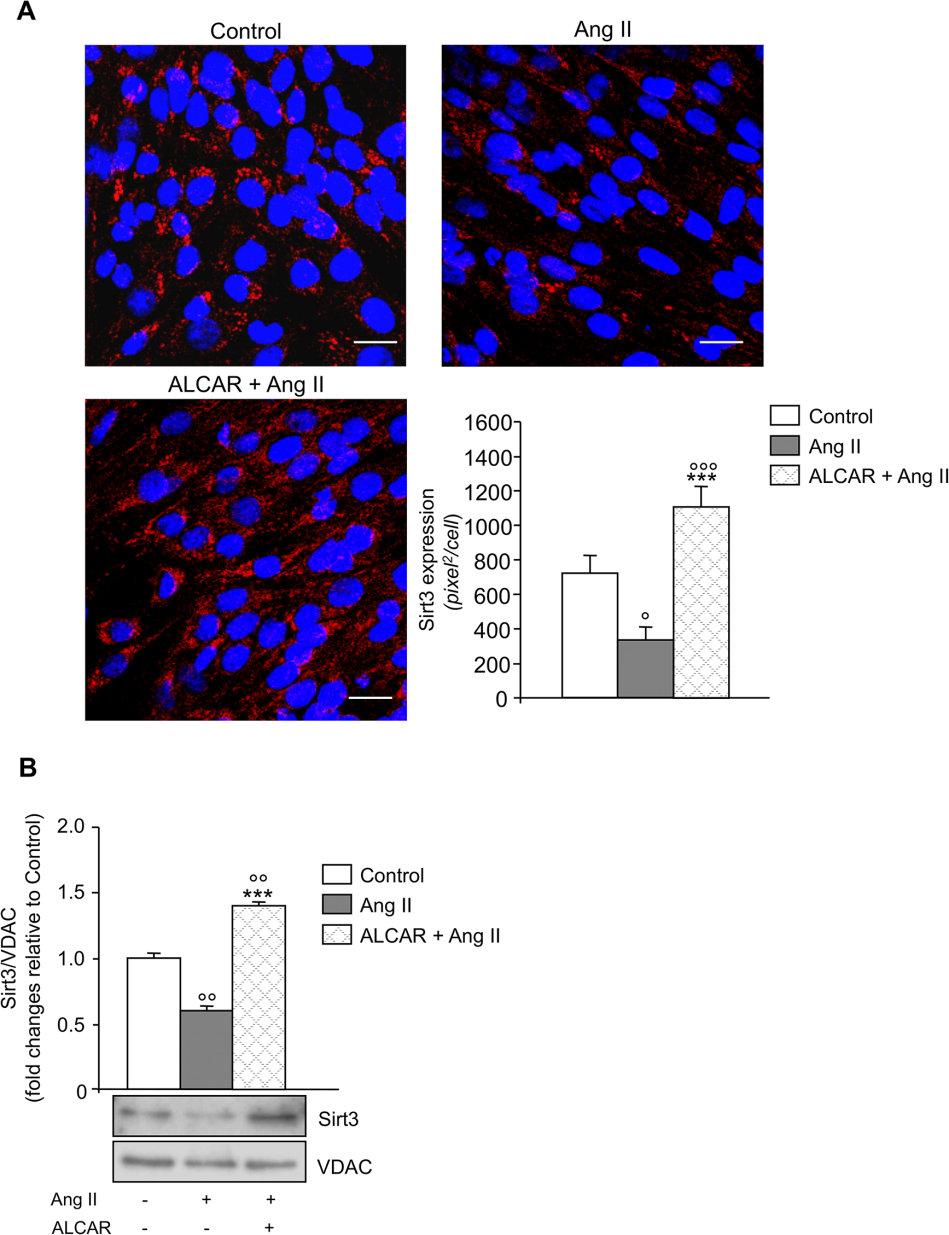
Ang II inhibits Sirt3 protein expression. (A) Representative images of Sirt3 immunofluorescence staining (red) in control and Ang II-treated L6 myotubes in the absence and presence of ALCAR. Nuclei were stained with DAPI (blue). Scale bars, 20 μm. Quantification of Sirt3 protein expression. Results are mean ± SE (n = 10). (B) Densitometric analysis (top) and representative western blot of Sirt3 (bottom) in mitochondria isolated from control and Ang II-treated L6 myotubes in the absence and presence of ALCAR. VDAC, mitochondria-loading protein. Results are mean ± SE (n = 3). °P < 0.05, °°P < 0.01, °°°P<0.001 vs. Control; ***P < 0.001 vs. Ang II.

**Fig 3:**
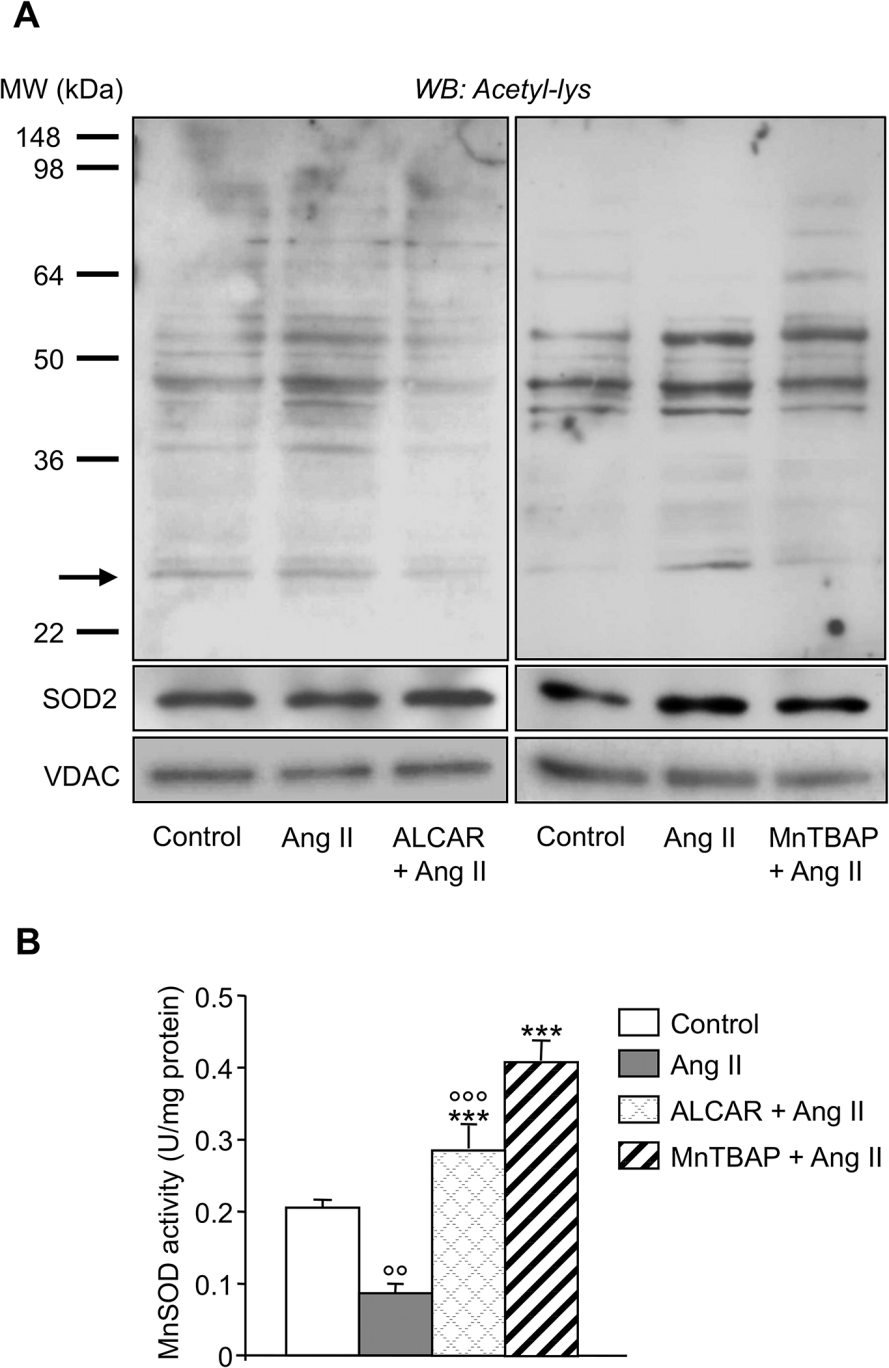
Ang II inhibits Sirt3 deacetylase activity and impairs mitochondrial antioxidant defense. (A) Western blot of acetylated proteins in mitochondria from control and Ang II-treated L6 myotubes in the absence and presence of ALCAR or MnTBAP. The arrow marks the band corresponding to MnSOD that was revealed on the same membrane after stripping and incubation with an anti-MnSOD specific antibody. Expression of the loading protein VDAC was analyzed by western blot in the same samples run in parallel. (B) MnSOD activity in isolated mitochondria. Results are mean ± SE (n = 5). °°P < 0.01,°°°P < 0.001 *vs*. Control; ***P < 0.001 *vs*. Ang II.

In physiological conditions the accumulation of ROS within mitochondria is counteracted by antioxidant enzymes whose activity is regulated by the NAD^+^-dependent mitochondrial deacetylase Sirt3 [20]. Ang II markedly reduced Sirt3 protein expression in L6 myotubes as revealed by lower immune reactive speckled perinuclear staining compared to unstimulated cells (Fig 2A). Consistently, Sirt3 protein content in isolated mitochondria was reduced by 50% in response to Ang II, while normal levels were found in the presence of ALCAR (Fig 2B). Ang II also affected Sirt3 deacetylase activity as seen in the evaluation of protein acetylation in mitochondria from unstimulated or Ang II-treated L6 myotubes. Ang II induced hyperacetylation of mitochondrial proteins with molecular weights of approximately 55, 48, 44, 38, and 26 kDa, the latter corresponding to MnSOD (Fig 3A). This effect was concomitant with the reduction of MnSOD antioxidant activity (Fig 3B). The addition of ALCAR reversed the inhibitory effect of Ang II on Sirt3 activity (Fig 3A, left) and restored the cell antioxidant defense, normalizing MnSOD activity (Fig 3B) and gene expression (S2 Fig) in L6 cells. Similarly, MnTBAP, by fostering MnSOD activity (Fig 3B), limited mitochondrial protein acetylation induced by Ang II in L6 myotubes (Fig 3A, right) confirming an inhibitory role for oxygen radicals in Sirt3-dependent mitochondrial protein deacetylation.

**Fig 4:**
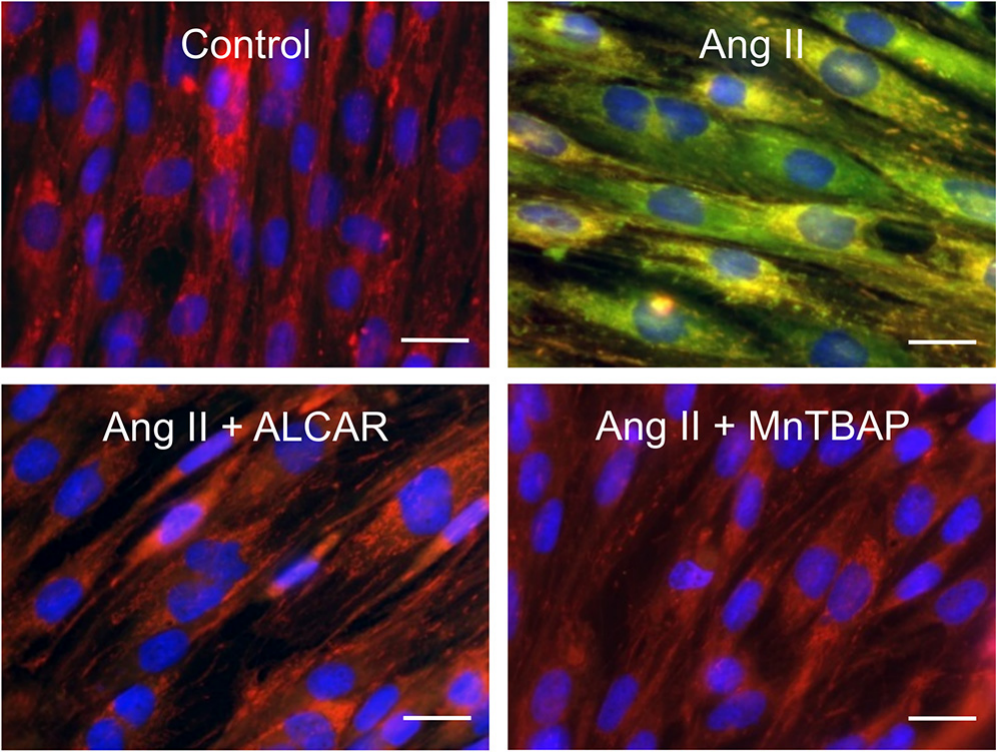
The mPTP opening contributes to Ang II-induced mitochondrial protein acetylation. Representative images of JC-1 staining show punctate orange-red fluorescence in polarized mitochondria from controls and Ang II-treated L6 myotubes in the presence of ALCAR or MnTBAP. Diffuse green fluorescence marks depolarized mitochondria in cells exposed to Ang II alone. Scale bars, 20 μm.

To assess whether impaired Sirt3 deacetylase activity induced by Ang II was due to the deleterious effect of mitochondrial O_2_^•^ opening the mitochondrial permeability transition pore (mPTP) leading to the collapse of ΔΨ, we investigated the effect of Ang II on ΔΨ -sensing dye JC-1 in both the absence and presence of ALCAR or MnTBAP. Ang II-treated L6 myotubes showed mitochondrial depolarization, as evidenced by diffuse cytoplasmic JC-1 green staining, that was prevented by both ALCAR and MnTBAP (Fig 4A–4D) suggesting mitochondrial O_2_^•^ as a mPTP trigger. To confirm the functional link between the mPTP opening and modulation of Sirt3 deacetylase activity, experiments were repeated in the presence of CsA, known to inhibit mPTP opening. Prevention of mitochondrial depolarization by CsA inhibited Ang II-induced mitochondrial protein acetylation, reflecting improved Sirt3 activity (S3 Fig).

### Ang II-induced Sirt3 dysfunction leads to impairment of AMPK/NAMPT signaling

**Fig 5:**
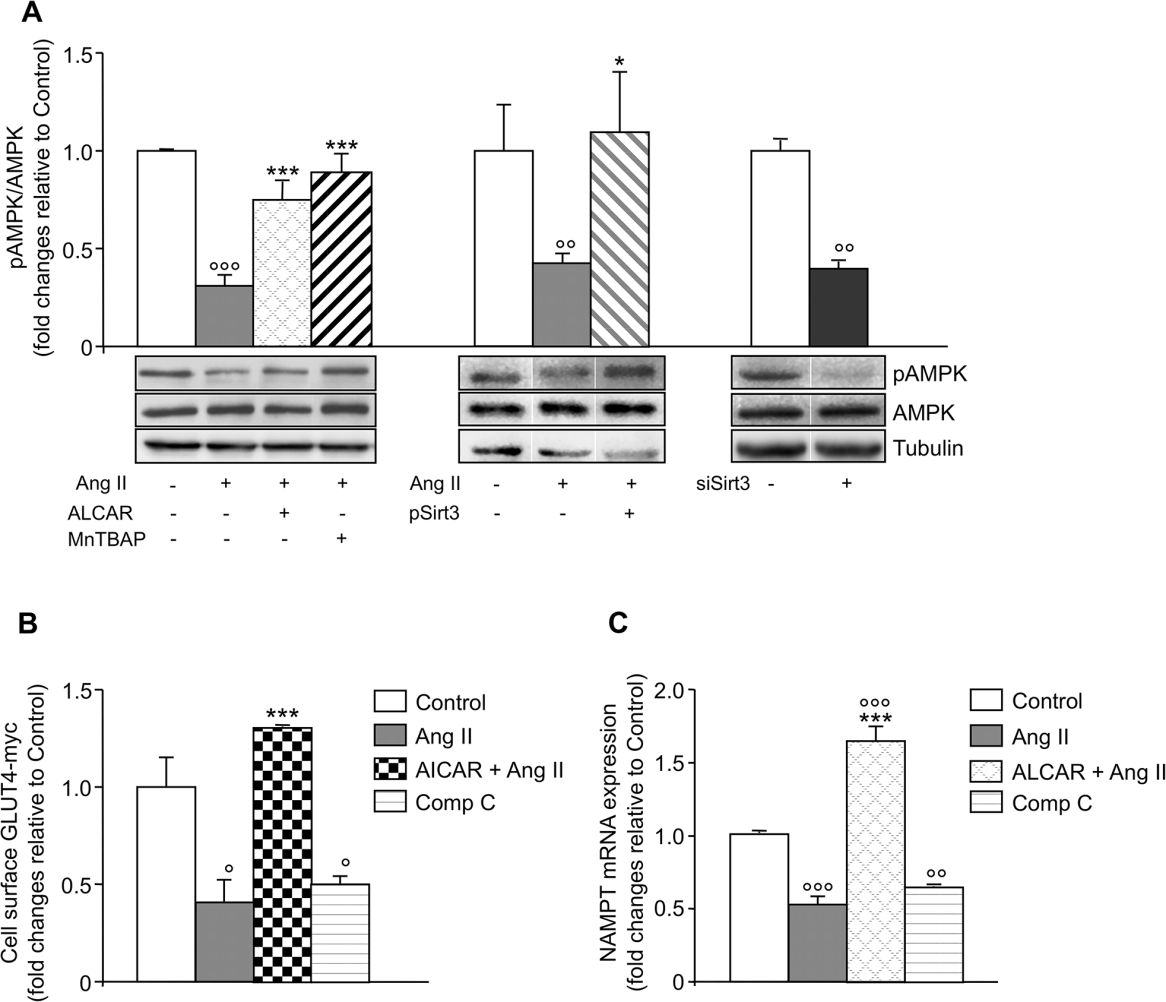
Ang II down-regulates AMPK/NAMPT signaling. (A) Densitometric analysis (top) and representative western blot (bottom) of pAMPK/total AMPK in control and Ang II-treated L6 myotubes in the absence and presence of ALCAR or MnTBAP (left); in Ang II-treated L6 myotubes untransfected and transfected with GFP-tagged Sirt3 plasmid (pSirt3) (middle); in irrelevant siRNA and siSirt3 transfected unstimulated L6 myotubes (right). Results are mean ± SE (n = 5, left; n = 3 middle and right, lanes were run on the same gel but were noncontiguous). (B) Surface GLUT4-myc density. Results are mean ± SE (n = 3). (C) Real time PCR of NAMPT mRNA. Results are mean ± SE (n = 3). °P < 0.05, °°P < 0.01, °°°P < 0.001 *vs*. Control; *P < 0.05, ***P < 0.001 *vs*. Ang II.

To elucidate the pathways linking Sirt3 dysfunction-induced by Ang II- to impaired GLUT4 translocation, we focused on AMPK, a master regulator of cellular energy metabolism involved in glucose transport in skeletal muscles [28, 29]. Ang II reduced the pAMPK/AMPK ratio by about 70% in L6 myotubes (Fig 5A, left). ALCAR normalized AMPK phosphorylation in Ang II-treated L6 myotubes (Fig 5A, left), while it did not affect the pAMPK/AMPK ratio in control cells (fold changes *vs*. control, ALCAR 0.99 ± 0.16 *vs*. 1.0). Scavenging mitochondrial O_2_^•^ by MnTBAP yielded similar results on AMPK phosphorylation in Ang II-treated and control L6 myotubes (Fig 5A, left). Activation of AMPK by AICAR [30] normalized GLUT4–myc levels on the surface of Ang II-treated cells (Fig 5B), while inhibition of AMPK by compound C [31] mimicked the inhibitory effect of Ang II on GLUT4 translocation (Fig 5B) suggesting a functional link between AMPK inactivation and impaired glucose transport.

To establish the role of Sirt3 in AMPK regulation we studied the effect of Ang II on the pAMPK/AMPK ratio in Sirt3 overexpressing cells (pSirt3). A 4-fold increase in Sirt3 protein expression (S4 Fig) abrogated Ang II-induced inactivation of AMPK (Fig 5A, middle). On the other hand, silencing Sirt3 with small interfering RNA (siSirt3) resulted in a 69% decrease of Sirt3 mRNA compared to scrambled cells (0.31 ± 0.05 vs. 1.0 ± 0.15, P < 0.05) and led to a significant reduction of the pAMPK/AMPK ratio (Fig 5A, right) confirming the regulatory effect of Sirt3 on AMPK.

In skeletal muscles AMPK is known to affect the expression of NAMPT, the rate-limiting enzyme in the NAD salvage pathway [32, 33]. Consistent with AMPK inhibition, Ang II down-regulated NAMPT mRNA levels that were normalized by ALCAR (Fig 5B). The inhibitory effect of Ang II on AMPK was comparable to that of compound C confirming AMPK as an upstream regulator of NAMPT in L6 myotubes (Fig 5C).

### ALCAR protection of skeletal muscle cells against Ang II-induced insulin resistance requires Sirt3

**Fig 6:**
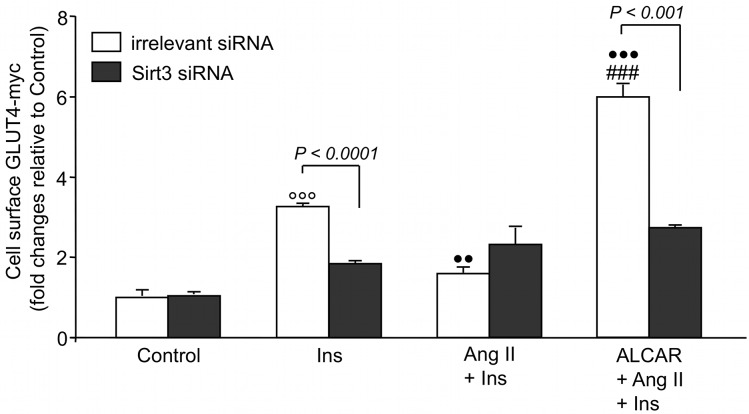
ALCAR protection of skeletal muscle cells against Ang II-induced insulin resistance requires Sirt3. L6 GLUT4-myc myotubes were transfected with Sirt3 siRNA or irrelevant siRNA and after 48 h scrambled controls or Sirt3 KD cells were incubated with Ang II for 24 h before and during 30-min stimulation with insulin. ALCAR was added 1 h before Ang II and maintained throughout the experiment. Results are mean ± SE (n = 3). L6 GLUT4-myc myotubes transfected with irrelevant siRNA: °°°P < 0.001 *vs*. Control; ••P < 0.01 and •••P < 0.001 *vs*. Ins; ###P < 0.001 *vs*. Ang II + Ins.

To further demonstrate the functional role of Sirt3 in the protective effect of ALCAR on Ang II-induced insulin resistance, we evaluated cell surface GLUT4 expression in Sirt3 siRNA and irrelevant siRNA-transfected L6 GLUT4-myc myotubes in the presence and absence of ALCAR. Sirt3 knocked-down cells showed a 70% decrease of Sirt3 mRNA compared to scrambled cells (0.31 ± 0.02 *vs*. 1.0 ± 0.01, P < 0.0001). As shown in Fig 6, insulin promoted a 3-fold increase in cell surface GLUT4-myc in irrelevant siRNA myotubes that was significantly reduced by Ang II. ALCAR prevented the inhibitory effect of Ang II on insulin-mediated GLUT4 transport in irrelevant siRNA cells (Fig 6). Silenced Sirt3 myotubes became resistant to insulin that failed to stimulate GLUT4-myc translocation to the cell surface (Fig 6). Importantly, knocking out Sirt3 negated the effect of Ang II as well as the protective action of ALCAR on GLUT4 translocation.

## Discussion

Here we found that 1) Ang II promotes insulin resistance through mitochondrial ROS, 2) enhanced mitochondrial O_2_^•^ production leads to Sirt3 dysfunction, impairing mitochondrial antioxidant defense, 3) ALCAR protects against Ang II-induced insulin resistance by preventing Sirt3 dysfunction.

The present results are consistent with data that chronic delivery of mitochondrial SOD mimetic improved glucose homeostasis in *ob/ob* mice [34] and that MnSOD transgenic mice were protected from high fat diet-induced insulin resistance [35]. Accumulated ROS contribute to mitochondrial dysfunction through the mPTP opening that depletes mitochondrial NAD^+^, the substrate for Sirt3 deacetylase activity [36]. Our findings that MnTBAP prevented Ang II-induced mitochondria depolarization and acetylation of mitochondrial proteins would indicate that O_2_^•^, by opening mPTP, leads to Sirt3 dysregulation, by activating a feed-forward loop that sustains oxidative stress in skeletal muscle cells. Previous evidence in cultured renal tubular epithelial cells of a link between Ang II and Sirt3 via Ang II type 1 receptor (AT1R) [21], suggests a possible role of AT1R in Ang II-induced Sirt3 dysfunction in the present setting.

Sirt3 activity can be regulated by AMPK through NAMPT, the rate-limiting enzyme in the biosynthesis of Sirt3 substrate NAD [37]. In this context, it is reported that AMPK signaling regulates NAMPT mRNA and protein expression in skeletal muscles [32, 33]. Our results showing that down-regulation of NAMPT was secondary to AMPK inhibition indicate that AMPK has a causative role in modulating NAMPT gene transcription, and possibly Sirt3 deacetylase activity in response to Ang II. AMPK regulates insulin action [38–40] and is a drug target for diabetes and metabolic syndrome [40–42]. When AMPK was inhibited by Ang II, there was reduced cell surface GLUT4 expression, which was reversed by the AMPK agonist AICAR. Our findings are in line with the evidence that Ang II inhibits AMPK-dependent glucose uptake in the soleus muscle [43] and that AMPK activation is part of the protective effect of angiotensin receptor blockade against Ang II-induced insulin resistance [44].

To add to the complexity, one might consider that excessive oxygen radical production also negatively regulates AMPK function. There is already evidence that AMPK can be activated by Sirt3 when it deacetylates LKB1 [45], the primary upstream kinase of AMPK. Moreover, skeletal muscles from Sirt3-deficient mice show reduced AMPK phosphorylation [46], while increased muscle AMPK activation is observed in transgenic mice with muscle-specific expression of the murine Sirt3 short isoform [47].

Previous studies in L6 rat skeletal muscle cells showed that Ang II impairs insulin signaling by inhibiting insulin-induced tyrosine phosphorylation of insulin receptor substrate 1 (IRS-1) and the activation of Akt [12]. Similarly, Sirt3 deletion in cultured myoblasts impairs insulin signaling, leading to a decrease in tyrosine phosphorylation of IRS-1 [48]. It is conceivable that Ang II-induced Sirt3 dysfunction in our setting negatively regulates insulin metabolic signaling, affecting both IRS-1 and the distal downstream step Akt activation.

Our study focused on mitochondrial ROS as a driver of Ang II-induced insulin resistance in skeletal muscle cells. However, NADPH oxidase has been also reported as a source of ROS induced by Ang II in L6 myotubes [12]. The relative role of NADPH oxidase and mitochondria in ROS generation in Ang II-treated skeletal muscle cells is unknown. There is emerging evidence of cross talk between NADPH oxidase and mitochondria in regulating ROS generation. In different settings, NADPH oxidase-derived ROS can trigger mitochondrial ROS formation and vice-versa [49–51]. It is conceivable that Ang II-induced NADPH oxidase activation would concur to trigger mitochondrial changes in L6 myotubes.

Disorders characterized by mitochondrial dysfunction and oxidative stress, such as neurodegeneration and cognitive deficit [52, 53], benefit from ALCAR supplementation, which seems to act as an antioxidant, likely by improving mitochondrial efficiency [54, 55]. In this study, we provide evidence that by preventing the inhibitory effect of Ang II on Sirt3 expression and activity, ALCAR restored the antioxidant activity of MnSOD, rescuing skeletal muscle cells from mitochondrial superoxide-driven insulin resistance. Here the beneficial effects of ALCAR in improving insulin sensitivity vanished when Ang II-treated myotubes were silenced for Sirt3, underlining that the antioxidant effects of ALCAR depend on Sirt3-mediated mitochondria protection. Our data showing that ALCAR normalized NAMPT expression through the activation of AMPK additionally support Sirt3 as a target for the insulin-sensitizing effect of ALCAR. That the compound may act on the AMPK pathway is also suggested by previous findings in rat skeletal muscle cells [56] and soleus muscles [57].

Thus, Sirt3 could be the unifying intracellular molecular signaling through which L-carnitine and its esters, including ALCAR, protect mitochondria and ameliorate insulin resistance. This is relevant in view of the emerging role of L-carnitine and its derivatives as promising treatment for diseases associated with mitochondrial dysfunction [58]. In this context propionyl-L-carnitine has been shown to improve mitochondrial respiratory chain activity in the livers of diet-induced obese mice and to protect these animals from insulin resistance and cardiovascular complications [59].

One limitation of the present findings is that they are gathered from cultured skeletal muscle cells. In vivo follow-up studies in experimental models of RAS-related insulin resistance are needed to definitely prove the functional relevance of these findings.

In conclusion, our data clarify and explain the Ang II intracellular molecular signaling that promotes insulin resistance in skeletal muscle cells through mitochondrial oxidative stress and Sirt3 dysfunction. It is conceivable that mechanism(s) at work in skeletal muscles may contribute to insulin resistance induced by Ang II in other tissues. The present study also highlights Sirt3 as a candidate therapeutic target for antioxidant and mitochondria-protective agents that counteract the deleterious effects of Ang II on insulin sensitivity and paves the way for testing novel treatments for insulin resistance, metabolic syndrome, and possibly diabetes, based on the pharmacological modulation of Sirt3.

## Supporting Information

S1 Fig Effect of Ang II on GLUT4 translocation in GLUT4-myc L6 myotubes. Results are mean ± SE (n = 6). °°°P < 0.001 *vs*. Control; ***P < 0.001 *vs*. Ang II.
(TIF)

S2 Fig Real time PCR of MnSOD mRNA. Results are mean ± SE (n = 5). °°°P < 0.001 *vs*. Control; ***P < 0.001 *vs*. Ang II.
(TIF)

S3 Fig Cyclosporin A prevents Ang II-induced mitochondrial depolarization and protein acetylation in L6 myotubes. (A) Representative image of JC-1 staining in mitochondria from Ang II-treated L6 myotubes in the presence of CsA showing puntate orange-red fluorescence (see Fig 4 for comparison). Scale bar, 20 μm. (B) Western blot of acetylated proteins in mitochondria from controls and Ang II-treated cells in the absence and presence of CsA.
(TIF)

S4 Fig Sirt3 plasmidic overexpression in L6 myotubes. (A) Representative images of staining for Sirt3 (red) and GFP (green) in L6 myotubes untransfected and transfected with a GFP-tagged Sirt3 plasmid (pSirt3). Scale bar, 20 μm. (B) Quantitative analysis of Sirt3 and GFP positive areas. Results are mean ± SE (n = 3). ***P < 0.001 *vs*. Control.
(TIF)
