# Supplementary figures and images for: Sirtuin3 Dysfunction Is the Key Determinant of Skeletal Muscle Insulin Resistance by Angiotensin II
Source: PLoS One. 2015 May 19;10(5):e0127172. doi: 10.1371/journal.pone.0127172 (PMC4437781; doi:10.1371/journal.pone.0127172)

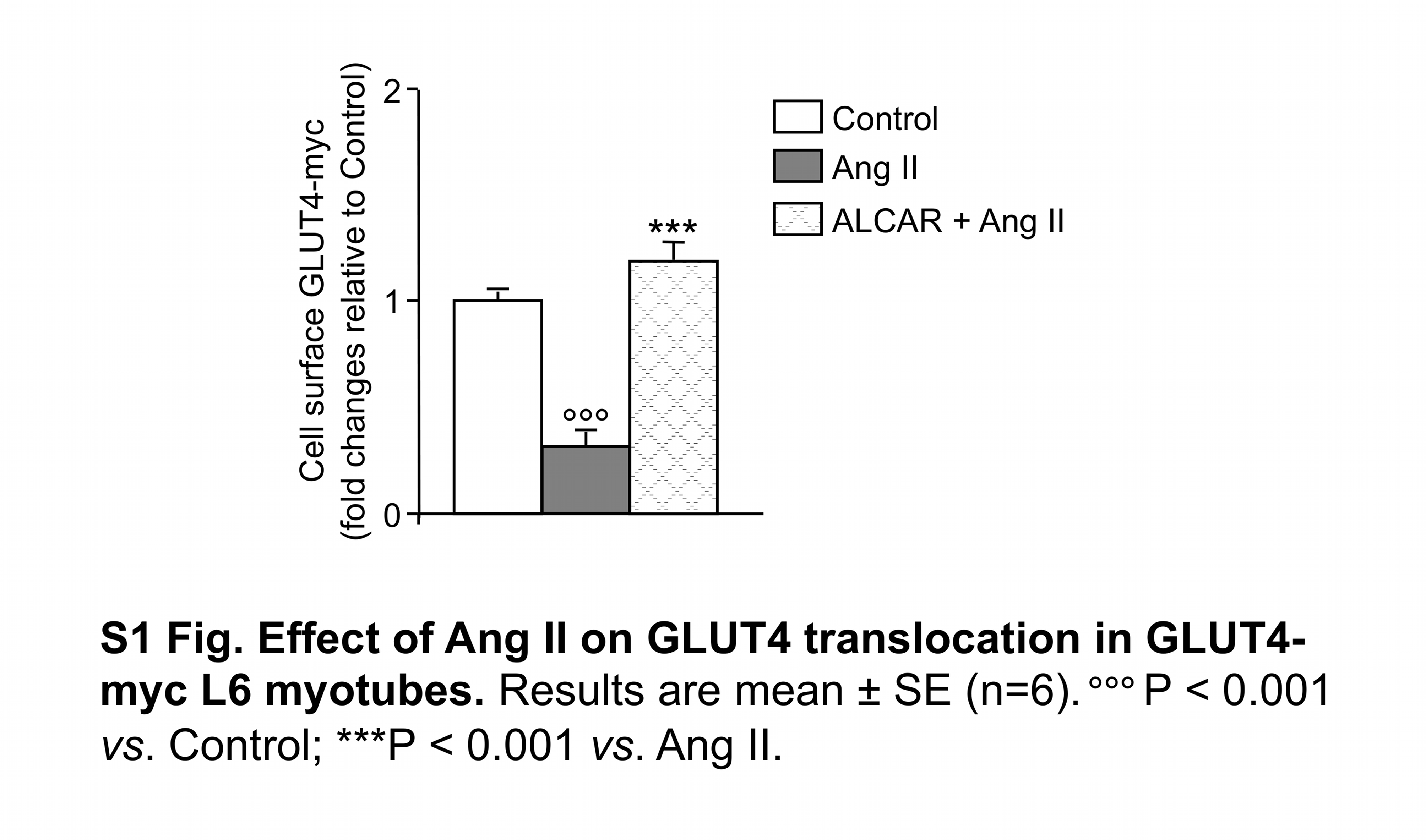

Supplement: S1 Fig — Results are mean ± SE (n = 6). °°°P < 0.001 vs. Control; ***P < 0.001 vs. Ang II. (TIF) [file pone.0127172.s001.tif]

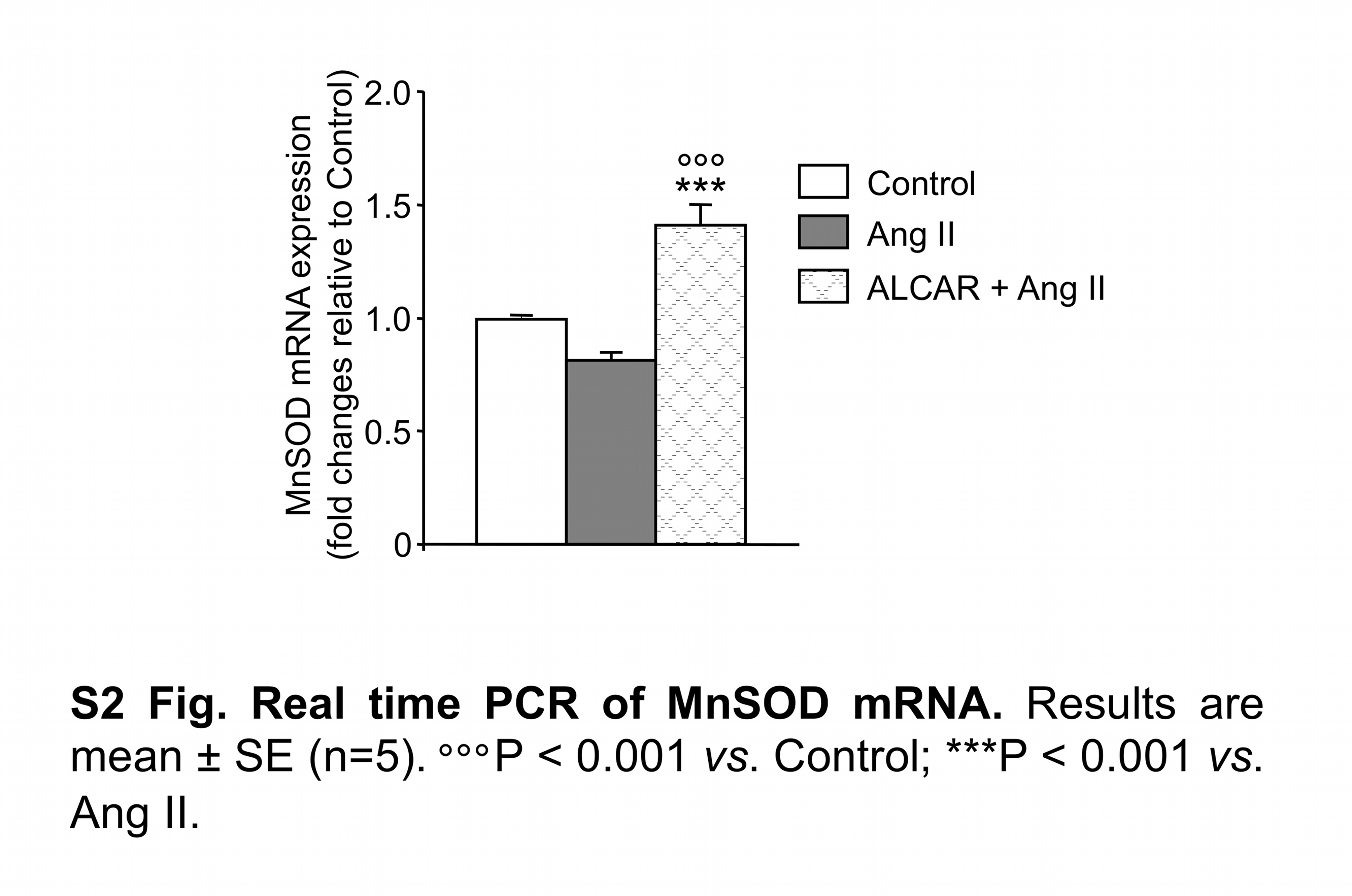

Supplement: S2 Fig — Results are mean ± SE (n = 5). °°°P < 0.001 vs. Control; ***P < 0.001 vs. Ang II. (TIF) [file pone.0127172.s002.tif]

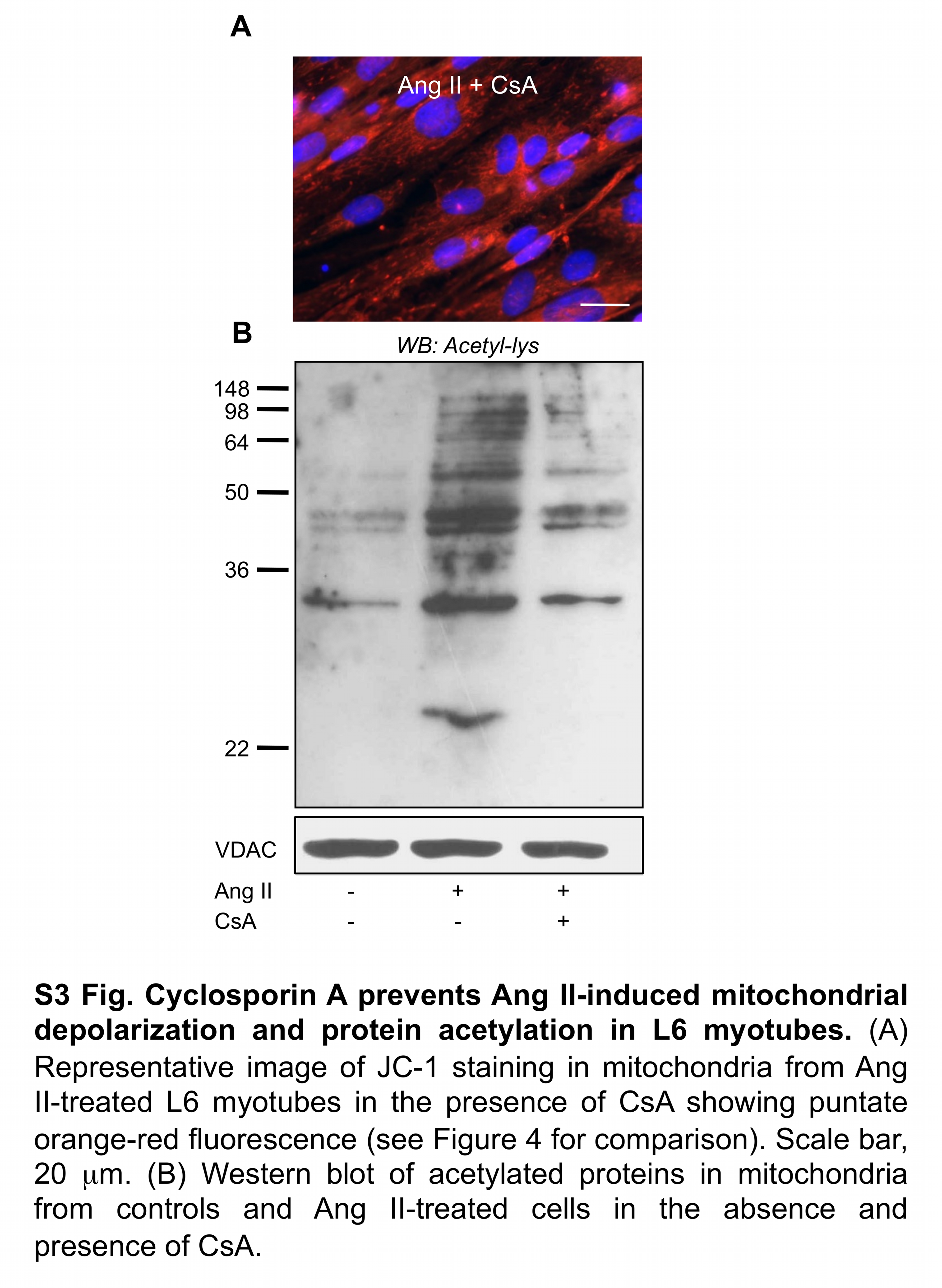

Supplement: S3 Fig — (A) Representative image of JC-1 staining in mitochondria from Ang II-treated L6 myotubes in the presence of CsA showing puntate orange-red fluorescence (see Fig 4 for comparison). Scale bar, 20 μm. (B) Western blot of acetylated proteins in mitochondria from controls and Ang II-treated cells in the absence and presence of CsA. (TIF) [file pone.0127172.s003.tif]

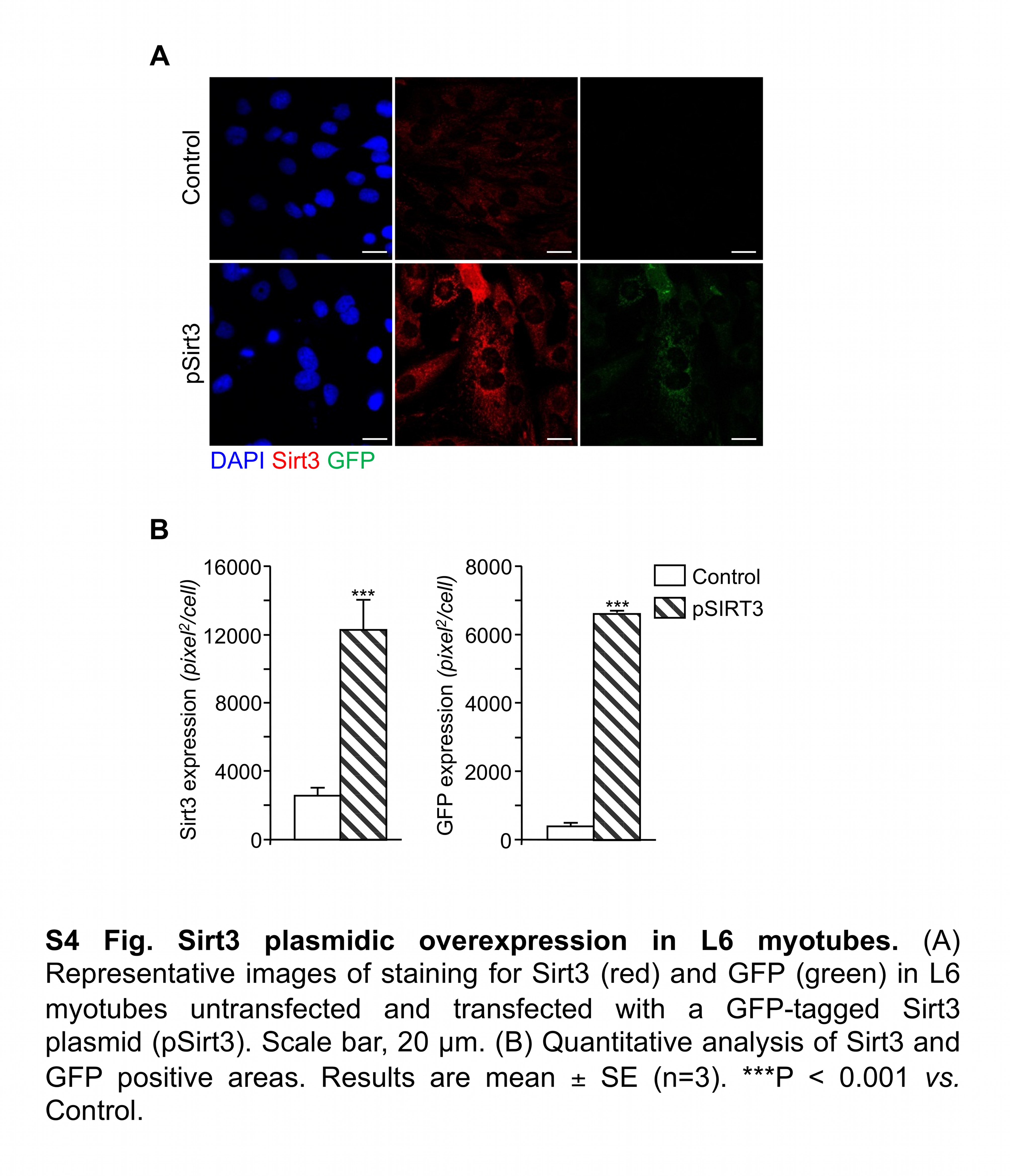

Supplement: S4 Fig — (A) Representative images of staining for Sirt3 (red) and GFP (green) in L6 myotubes untransfected and transfected with a GFP-tagged Sirt3 plasmid (pSirt3). Scale bar, 20 μm. (B) Quantitative analysis of Sirt3 and GFP positive areas. Results are mean ± SE (n = 3). ***P < 0.001 vs. Control. (TIF) [file pone.0127172.s004.tif]
